# QR-Code-Integrated Colorimetric Sensors for Simultaneous Temperature Threshold and Oxygen Detection

**DOI:** 10.3390/s26206496

**Published:** 2026-10-14

**Authors:** Carmen López-León, César Moyano, Víctor Toral, Diego P. Morales, Alfonso Salinas-Castillo, Pablo Salcedo-Abraira, Francisco J. Romero

**Affiliations:** 1 Department of Analytical Chemistry, Faculty of Sciences, University of Granada, Av. Fuente Nueva s/n, 18071 Granada, Spain; carmenll@ugr.es (C.L.-L.); alfonsos@ugr.es (A.S.-C.); 2 Spica BioTech SL, Calle Pintor Maldonado 23 (9), 18007 Granada, Spain; info@spicabiotech.com; 3 Department of Electronics and Computer Technology, Faculty of Sciences, University of Granada, Av. Fuente Nueva s/n, 18071 Granada, Spain; vtoral@ugr.es (V.T.); diegopm@ugr.es (D.P.M.); 4 Department of Inorganic Chemistry, Faculty of Sciences, University of Granada, Av. Fuente Nueva s/n, 18071 Granada, Spain; psalcedo@ugr.es

**Keywords:** QR codes, colorimetric sensors, thermochromic materials, oxygen sensors, multi-analyte sensing, smart packaging

## Abstract

Sensor-integrated Quick Response (QR) codes are a promising route for combining product traceability with sensing capabilities. However, most reported platforms rely on binary read/no-read mechanisms in which sensor activation affects QR readability. To overcome this, we propose a QR architecture that preserves the information-storage and decoding functions of the code while enabling simultaneous environmental monitoring. The approach exploits the intrinsic error-correction capability of QR codes by replacing specific modules of a QR code (with error-correction level H) with colorimetric sensing elements, thereby decoupling sensor response from QR readability. A thermochromic polydimethylsiloxane (PDMS) composite and a photoactivatable ZnO-alginate hydrogel containing methylene blue (MB) were integrated into the QR structure to simultaneously detect a temperature threshold and oxygen presence while maintaining decoding functionality. Both sensors exhibit red-channel responses centered at 610–660 nm, enabling grayscale-based image analysis. A smartphone-based workflow combining red-channel extraction, contrast correction and binarization decodes the embedded identifiers and detects sensor activation through module-level comparison. In a smart-packaging demonstration, the platform successfully identified different combinations of refrigeration and vacuum-sealing states. Furthermore, sensor layouts can be uniquely linked to the encoded identifier, providing additional tamper resistance, traceability, and product authentication. The proposed strategy establishes a versatile and low-cost platform for multiparameter monitoring within existing QR-based infrastructures.

## 1. Introduction

Nowadays, access to accurate and real-time information has become essential in modern society, particularly in areas related to health, safety, product quality, and logistics. Continuous monitoring of environmental and product-related parameters is increasingly important in sectors such as food packaging, pharmaceutical distribution, supply-chain management, and environmental control, where product quality and safety depend strongly on the conditions experienced during multiple phases (production, storage, transportation, and delivery) [1,2,3]. In this context, the emergence of new technological paradigms, including the Internet of Things (IoT), cloud computing, and artificial intelligence (AI), has enabled the development of intelligent monitoring systems capable of collecting, transmitting, and processing information throughout the entire lifecycle of a product [4,5]. These technologies facilitate real-time decision making, improve traceability, and enable early detection of potentially harmful deviations from required environmental conditions [6].

In the context of logistics, QR (Quick Response) codes have proven to be an effective solution for connecting physical products with digital information. Their low cost, ease of generation, high information density, and ability to be scanned using conventional smartphone cameras make them attractive tools for product identification and traceability [7,8]. Furthermore, the widespread adoption of smartphones has further established QR codes as a convenient access point to IoT infrastructures, allowing virtually any user to retrieve product-related information instantaneously [9]. In their conventional implementation, QR codes act as passive carriers of static information, providing access to datasets such as product identifiers, batch numbers, manufacturing records, logistics information, or web-based databases [10,11,12].

At the same time, advances in the field of integrated sensors and detection systems have opened new possibilities for monitoring environmental conditions directly at the product level. Modern sensing materials can modify their physical, chemical, or optical properties in response to external stimuli such as gases, light, temperature, humidity, or pH variations [13,14]. Among the available sensing technologies, solid-state colorimetric sensors have attracted significant attention in recent years due to their simplicity, low manufacturing cost, visual readability, and compatibility with portable image-acquisition devices. In these systems, the interaction between the analyte and the sensing material induces a measurable color change that can be detected visually or quantified through digital image processing [15,16]. Typically, color information extracted from RGB images is converted into analytical signals that provide quantitative information about the monitored parameter [17,18].

The complementary characteristics of QR codes and colorimetric sensors have motivated the development of sensor-integrated QR platforms, in which specific regions of the QR code are modified to respond to environmental stimuli [8,19,20,21]. As shown in Table 1, the combination of QR codes and colorimetric sensors has been successfully applied for the monitoring of parameters such as temperature [22,23,24,25,26,27,28,29], pH, volatile nitrogen compounds associated with food spoilage [30], relative humidity [31], as well as microbiological contamination by *Escherichia coli* [32]. Moreover, diverse approaches have been employed for their fabrication. For instance, temperature-sensitive QR systems have been developed using luminescent nanoparticles [22,23], thermochromic inks [24,25,26], and hydrogel-based materials [27,28,33], while gas-sensitive devices have been based on immobilized colorimetric dyes and flexible printed platforms [31,34]. Many of these systems rely on a binary “read/no-read” mechanism, where the sensor response directly affects QR readability.

Despite these advances, several important limitations remain. First, most QR-based sensing platforms are designed for the detection of a single analyte, despite many practical applications require simultaneous monitoring of multiple environmental parameters [35,36]. Second, systems based on QR readability loss may lead to ambiguous results, since unsuccessful decoding can originate not only from the sensing event but also from inadequate illumination, poor image acquisition, or defects in the code itself [8]. Third, the use of optical calibration and illumination-correction strategies remains limited, despite their importance for reliable smartphone-based measurements under real-world conditions [34,37].

For these reasons, increasing attention has recently been devoted to multi-analyte sensing systems capable of detecting several parameters simultaneously within a single QR platform. Such an approach is particularly attractive for applications such as food quality control, environmental monitoring, and biomedical diagnostics, where multiple variables contribute to the overall state of the monitored system [8,38]. In these devices, the QR code not only encodes static information but also incorporates dynamic sensing regions that provide analyte-specific responses while preserving the functionality of the code [39].

In this work, we propose a sensor-integrated QR architecture that exploits the intrinsic error-correction capability of QR codes by replacing selected redundant data modules with colorimetric sensing elements. In this way, the sensor response is decoupled from QR readability, so that the code permanently preserves its static digital information while monitoring environmental conditions. Moreover, since the position of the sensing regions can be linked to the encoded identifier, the sensor layout provides an additional layer of traceability and authentication [40,41]. In addition, to address the limited use of illumination-correction strategies, the proposed workflow applies a percentile-based white/black normalization in which the printed QR modules act as internal references, compensating for variations in ambient lighting and smartphone cameras.

As a proof of concept, temperature and oxygen were selected as target parameters, since cold-chain maintenance and oxygen exclusion are essential, e.g., to preserve the quality of packaged food and pharmaceutical products [42,43,44]. Temperature sensing was implemented using a thermochromic polydimethylsiloxane (PDMS) composite with a transition temperature of 13 °C [18], and oxygen sensing using a photoactivatable alginate hydrogel containing ZnO nanoparticles and methylene blue (MB) [45]. These two sensors, based on different physicochemical principles, demonstrate the versatility of the proposed platform for simultaneous product traceability and environmental monitoring using a single smartphone-readable QR code.

## 2. Materials and Methods

### 2.1. Reagents and Equipment

For the thermochromic sensor, SYLGARD 184 silicone elastomer based on polydimethylsiloxane (PDMS) was purchased from Dow Inc. (Midland, MI, USA). The thermochromic pigment was obtained from Hali Industrial Co., Ltd. (Changzhou, China). These thermochromic pigments change color to colorless (reversible) when a certain temperature threshold is reached. In particular, we selected the color blue with a transition temperature of 13 °C, below that temperature the sensor acquires its blue color.

With regard to the oxygen sensor, the reagents sodium alginate (sample W201502), basic zinc carbonate (ZnCO_3_) ([ZnCO_3_]_2_·[Zn(OH)_2_]_3_), D-(+)-Glucono-1,5-lactone (GDL) (C_6_H_10_O_6_), glycerol 99.5% and methylene blue were purchased from Sigma Aldrich (Madrid, Spain). Zinc oxide (ZnO) nanoparticles were obtained from Scharlab S.L. (Barcelona, Spain). All chemical reagents were used as received. To vacuum pack the sensor, oxygen-absorbing packets (Ktxaby, Beijing, China) and a vacuum sealing machine (Bonsen Electronics Ltd., Dongguan, China) were used. To activate the oxygen sensor membranes, a UV LED lamp (λ = 365 nm, power 100 W, model ELE-PG365-100W from Elengated, Shenzhen, China) was used. The sensors were deposited in molds made from standard polylactic acid (PLA) filaments using a BQ WITBOX 2 3D printer (from BQ, Madrid, Spain).

### 2.2. Materials Characterization

Fourier-transform infrared spectra were obtained using a Bruker Tensor 27 spectrophotometer (Bruker Optik GmbH, Ettlingen, Germany) operating in attenuated total reflectance (ATR) mode. Ultraviolet–visible (UV–Vis) measurements were carried out on a Cary 5E UV–Vis–NIR spectrophotometer (Agilent Technologies Inc., Santa Clara, CA, USA) equipped with a Praying Mantis diffuse reflectance accessory. Spectra were recorded in the 300–800 nm range at room temperature using solid samples. Powder X-Ray diffraction was performed using a Bruker D8 DISCOVER (Bruker Optik GmbH, Ettlingen, Germany) equipped with CuKα radiation (λ = 1.54059 Å) in a 2θ range of 20–80° with a 0.02° step size and 40 s of acquisition time. To quantitatively evaluate the impact of the processing steps on the nanostructure, the average crystallite size (D) of the ZnO nanoparticles before and after entrapment within the alginate matrix was estimated using the Debye–Scherrer equation (D = Kλ/(β cos θ), where K = 0.9 is the shape factor, λ = 0.15406 nm is the Cu Kα wavelength, β is the full width at half maximum (FWHM) of the diffraction peak in radians, and θ is the Bragg angle [46].

### 2.3. Thermoresponsive Polymer Fabrication

PDMS was prepared using a 9:1 weight ratio between the base prepolymer (Sylgard 184 Silicone Elastomer Base from Dow Inc., Midland, MI, USA) and the corresponding curing agent (Sylgard 184 Curing Agent, from the same manufacturer). The mixture was stirred for approximately 10 min to ensure homogeneous dispersion. Subsequently, 150 mg of the thermochromic pigment (transition temperature: 13 °C) was incorporated and stirred for an additional 5 min until a uniform dispersion was achieved. The resulting mixture was poured into a Petri dish and subjected to vacuum for 40 min to remove air bubbles generated during the mixing process. The material was then poured into a 3D-printed mold made of PLA (sensor well: 17 × 17 × 2 mm) and cured in an oven at 100 °C for 1 h to obtain the final device. The cured material exhibits a reversible color change, appearing blue below 13 °C and becoming transparent at higher temperatures [18].

For UV–Vis characterization below the thermochromic transition temperature, an equivalent thermochromic pigment supplied by the same manufacturer was employed. In this case, a formulation with a transition temperature of 31 °C was selected and incorporated into the PDMS matrix following the same preparation protocol described above, which enabled spectral measurements at room temperature while maintaining the pigment in its colored state. As characterized in our previous work [18], both the 13 °C and 31 °C variants share the same composition: a lactone-type leuco dye as chromophore, dissolved in a fatty ester solvent and encapsulated in a melamine–formaldehyde resin. In these systems, the transition temperature is determined by the melting point of the solvent, so both variants differ mainly in the solvent formulation while sharing the same colored-state chromophore.

### 2.4. Oxygen-Responsive Hydrogel Fabrication

The oxygen-responsive hydrogel was prepared by dispersing ZnO (100 mg) and basic zinc carbonate (90 mg) nanoparticles in 30 mL of deionized water, followed by sonication at 60 °C until a homogeneous suspension was obtained. Sodium alginate (300 mg) was then added and dissolved under continuous stirring for 30 min. Glycerol (7.5 mL) was introduced as a plasticizer, followed by the addition of 1 mg of methylene blue as a redox indicator. The mixture was cooled to approximately 5 °C for 30 min to stabilize the system prior to gelation. Subsequently, 290 mg of GDL was added to gradually decrease the pH and induce ionic crosslinking of the alginate network via the release of Zn^2+^ ions. The solution was poured into a PLA mold (sensor well: 10 × 10 × 5 mm) and left to gel at 25 °C for 24 h. The resulting hydrogels were stored under refrigerated conditions until use [45].

The fabricated hydrogel exhibits a reversible colorimetric response to oxygen. Under atmospheric conditions, the sensor shows an intense blue coloration due to the oxidized form of methylene blue. Ultraviolet irradiation is used to photochemically reduce methylene blue to its leuco (colorless) form in the presence of suitable electron donors within the hydrogel matrix. This activation step was performed at room temperature after vacuum-sealing the sensor, by placing it at a distance of approximately 15 cm from the UV light source for 90 min, as illustrated in Figure S1. It should be noted that the commercial food-grade film used for vacuum packaging is sufficiently transparent in the near-UV range to allow the photoactivation of the sensor through the barrier layer, as evidenced by the complete decoloration of the methylene blue indicator after UV irradiation of the sealed package.

### 2.5. Colorimetric Characterization

The colorimetric characterization of both sensors was performed inside a CTC256 climate chamber (Memmert GmbH + Co. KG, Schwabach, Germany), which remained light-tight during measurements. Image acquisition was carried out using a 16 MP digital microscope (Dcorn, Shenzhen, China) placed at a vertical distance of 30 cm from the sensor. The sensors were illuminated with a dual white LED light source to ensure consistent imaging conditions during the whole characterization process.

For the thermoresponsive sensors, temperature-dependent measurements were conducted over a range of 2–40 °C across multiple heating–cooling cycles. Temperature profiles were programmed and monitored using Memmert Celsius software (version 10.1.6). To evaluate the dynamic response of the temperature sensor under realistic conditions, the temperature sensors were also stored in a refrigerator at 4 °C and then transferred to ambient temperature (27 °C), while their color change was continuously recorded. For oxygen-responsive sensors, vacuum-activated and UV-irradiated samples were removed from the oxygen-free environment and immediately placed under ambient conditions. Continuous image acquisition was used to monitor the color recovery process in real time. Periodic image acquisition for color characterization and processing was automated using Python 3.12.

Contrast correction was performed by white/black (W/B) normalization according to Equation (1) [38]:

(1) $$R_{C}=\frac{255}{\left(R_{W}-R_{B}\right)}\cdot \left(R_{\mathrm{raw}}-R_{B}\right)$$

where *R*_C_ is the red channel corrected value, *R*_raw_ is the raw value of the red channel of the acquired image and *R*_B_ and *R*_W_ are the black and white references, respectively.

Binarization was carried out according to Equation (2) [41]:

(2) Rbin=0,if RC≤GTH255,otherwise

where *G*_TH_ is the gray threshold value.

## 3. Results

### 3.1. Fourier Transform Infrared Spectroscopy (FTIR) Spectroscopy

**Figure 1:**
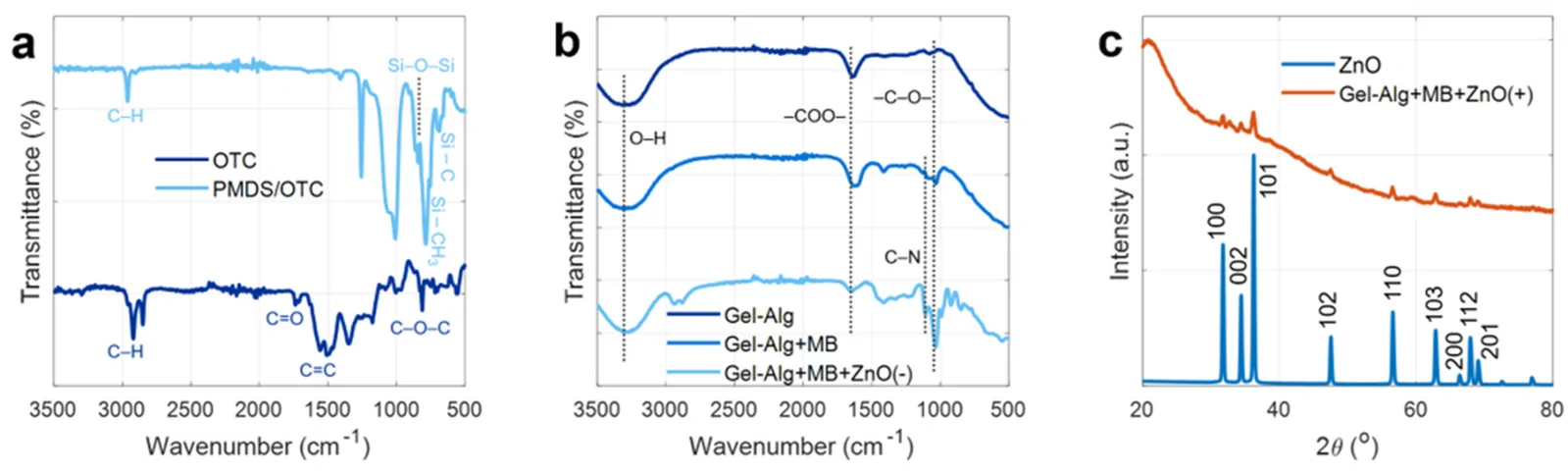
FTIR spectra of OTC and PDMS/OTC (**a**), and the different hydrogels (**b**). (**c**) XRD patterns of ZnO and Gel-Alg+MB+ZnO(+).

The FTIR spectra of all synthesized materials are presented in Figure 1a,b. The PDMS spectrum is dominated by the intense asymmetric Si–O–Si stretching vibration at 1010–1100 cm^−1^, accompanied by Si–O–Si symmetric stretching and Si–C stretching at 800 cm^−1^, Si–CH_3_ symmetric bending at 1260 cm^−1^, and C–H stretching of the methyl groups at 3000 cm^−1^ [47,48,49].

According to the manufacturer, the organic thermochromic pigment (OTC) is based on a lactone-type leuco dye; therefore, enhanced absorption at 1600–1750 cm^−1^ is consistent with C=O stretching of the lactone group in its closed, colorless ring form, along with aromatic C=C stretching. Bands around 1150–1250 cm^−1^ include contributions from C–O–C stretching of the lactone ring, overlapping with the Si–O–Si region of the matrix. The absence of new covalent bonds confirms that the pigment is physically embedded within the silicone network [18].

Additionally, as shown in the FTIR spectra of Gel-Alg, Gel-Alg+MB and Gel-ZnO(-) (Figure 1b), the alginate gel spectrum exhibits a broad O–H stretching band at 3300 cm^−1^ arising from the hydroxyl groups of the backbone and the water molecules present in the hydrogel, asymmetric and symmetric –COO^−^ stretching at 1600 cm^−1^ and 1400 cm^−1^ respectively, and C–O–C glycosidic and pyranose ring stretching in the 1000–1100 cm^−1^ region, these recognizable peak shifts can be attributed to the H-bonding resulting from the intermolecular interactions of alginate molecules and the crosslinking of alginate with the divalent Zn^2+^ cation [50,51]. This evidence indicates the realization of hydrogel gelation based on alginate and Zn^2+^, which causes changes in the molecular dynamics of the constituents. Upon incorporation of methylene blue, the Gel-Alg+MB spectrum closely resembles that of the bare hydrogel, with a subtle broadening around 1480–1600 cm^−1^ reflects the superimposition of the MB aromatic C=C and C=N stretching modes of its phenothiazine ring system onto the carboxylate bands of alginate, confirming successful dye loading without disruption of the polymer network. Additionally, a shift in the asymmetric –COO^−^ stretching band relative to the bare gel suggests coordination interactions between the carboxylate groups of alginate and the ZnO nanoparticle surface, indicative of interfacial bonding between the polymer and the inorganic filler [51,52,53,54,55]. These coordination interactions between the carboxylate groups of alginate and the surface Zn^2+^ sites of the ZnO nanoparticles may act as additional physical cross-linking points, complementary to the ionic cross-links formed by the Zn^2+^ ions released during gelation. Such interfacial bonding is expected to restrict the mobility of the polymer chains and increase the effective cross-linking density of the network, thus contributing to its mechanical integrity and limiting swelling, as reported for other ZnO–alginate composites [56].

### 3.2. Powder X-Ray Diffraction (PXRD) Analysis

The crystalline structure of the materials was analyzed by powder X-ray diffraction (Figure 1c). The diffractogram of bare ZnO shows a set of well-defined, sharp diffraction peaks at 2θ = 31.8°, 34.4°, 36.3°, 47.6°, 56.6°, 62.9°, 66.4°, 68.0° and 69.1°, which are fully indexed to the hexagonal wurtzite crystal structure of ZnO (JCPDS card No.01-089-0150), in agreement with previously reported results for ZnO nanoparticles [50,55,57]. The Miller indices (100), (002), (101), (102), (110), (103), (200), (112) and (201) correspond well to the characteristic reflections of the wurtzite phase [50,57,58,59]. The sharpness and intensity of these peaks confirm a high degree of crystallinity of the ZnO nanoparticles. In the diffractogram of Gel-Alg+MB+ZnO(+), the characteristic peaks of the wurtzite phase are preserved, confirming that the crystalline structure of ZnO is maintained upon incorporation into the hydrogel matrix. From the (101) reflection at 2θ = 36.3°, the average crystallite size was 26.4 nm for the pristine ZnO nanoparticles and 25.6 nm for the nanoparticles entrapped within the hydrogel. This minimal difference indicates that the crystalline domains of the wurtzite phase are preserved and that no significant crystal growth occurred during the sonication and gelation steps. Furthermore, the diffractogram of the composite also shows a broad, diffuse scattering background underlying the ZnO reflections, which is characteristic of the amorphous nature of the alginate hydrogel matrix. This amorphous halo, absent in pure ZnO, is consistent with the lack of long-range order in the cross-linked polysaccharide network. It should be noted that no additional diffraction peaks attributable to new crystalline phases are observed, indicating that neither the incorporation of methylene blue nor the gelation process induces any structural transformation of the ZnO nanoparticles. PXRD measurements were performed on both the hydrogel with the highest ZnO(+) concentration and the one with the lowest ZnO(-) concentration. We refer only to ZnO(+) since the sample with the lower concentration ZnO(-) exhibited an equivalent diffraction pattern, with ZnO peaks present but of lower relative intensity, confirming that the crystalline phase of the nanoparticles is not affected by their concentration within the hydrogel.

### 3.3. UV-Visible Spectroscopy Analysis

**Figure 2:**
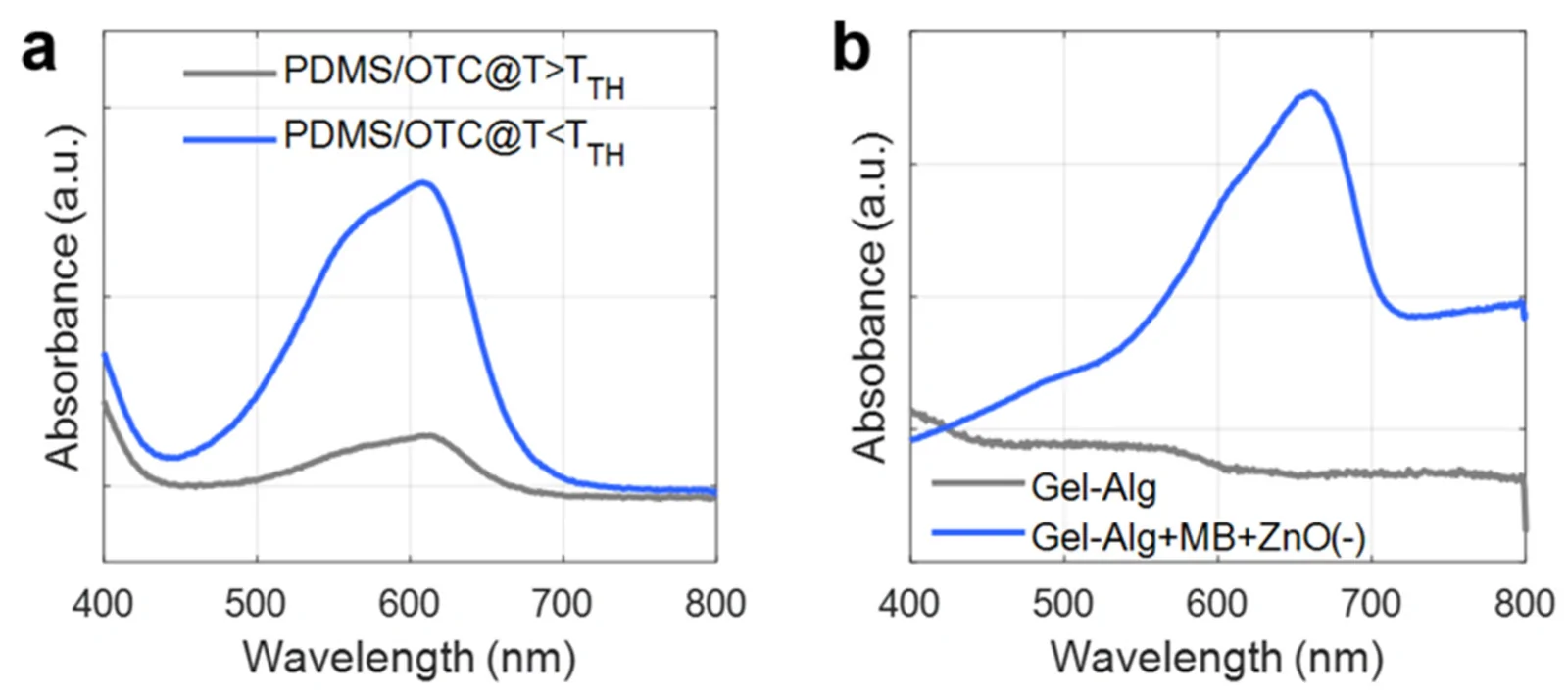
UV-Visible absorption of (**a**) PDMS/OTC and (**b**) respective hydrogels.

The optical properties of the thermochromic composite and the hydrogel system were evaluated by UV–visible spectroscopy, as shown in Figure 2. The spectrum of PDMS/OTC recorded above the thermochromic transition temperature (T > T_TH_ = 13 °C) shows a flat, featureless profile across the 400–800 nm range, consistent with the colorless, transparent state of the leuco dye in its closed lactone form (Figure 2a). In contrast, the spectrum of the 31 °C formulation, which remains in its colored state at room temperature, shows a well-defined absorption band with a maximum at 610 nm, corresponding to the open, colored form of the leuco dye. Since both formulations share the same chromophore, this band is representative of the colored state of the 13 °C sensor below T_TH_, as also reflected by the colorimetric characterization reported in Section 3.4.1. Furthermore, this appearance and disappearance of the absorption band is reversible, which directly reflects the thermochromic switching mechanism of the pigment and confirms its successful integration into the PDMS matrix without degradation of its optical response.

Regarding the hydrogel system (Figure 2b), the spectrum of Gel-Alg exhibits a relatively flat profile across the visible region, with no distinct absorption bands. This behavior is consistent with the transparent nature of sodium alginate, which lacks chromophoric groups capable of significant absorption in the visible range. Consequently, the alginate matrix itself does not contribute significantly to the optical response of the sensing system. In contrast, Gel-Alg+MB+ZnO(−) displays a pronounced absorption band centered at 655 nm, attributable to the π→π* electronic transition of methylene blue in its oxidized form [60,61]. Compared with the monomer band of MB in aqueous solution (~664 nm), this maximum shows a slight hypsochromic shift (~9 nm), which can be attributed to the interaction of the cationic dye with the anionic alginate network and the ZnO surface, favoring partial dye aggregation [62]. However, this minor shift does not affect the optical readout, since the absorption band remains within the 610–660 nm region analyzed by the red-channel image processing. The presence of this band confirms the successful incorporation of MB into the hydrogel and provides the optical activity required for the colorimetric oxygen-sensing mechanism. The alginate matrix thus acts as a transparent, optically neutral support, while the ZnO nanoparticles and methylene blue together constitute the photoactive and sensing components of the system. Furthermore, this absorption band serves as a useful optical marker for monitoring the redox transitions of methylene blue during the photocatalytic sensing process, which is fundamental for the reversible oxygen detection mechanism of the system.

These results demonstrate that the dominant absorption bands of both sensing systems lie within the red region of the visible spectrum (610–660 nm), which has direct implications for image-based colorimetric analysis. As it will also be shown in the colorimetric characterization, the blue-colored states of both sensors arise from strong attenuation of red wavelengths, whereas the transition to the colorless state results in a marked increase in red light transmission or reflectance. This effect is therefore highly advantageous for the use of red-channel grayscale analysis in subsequent image processing, particularly in view of the intended integration of the sensors into black-and-white QR codes, which rely on grayscale binarization.

### 3.4. Colorimetric Response

#### 3.4.1. Thermoresponsive Sensor

**Figure 3:**
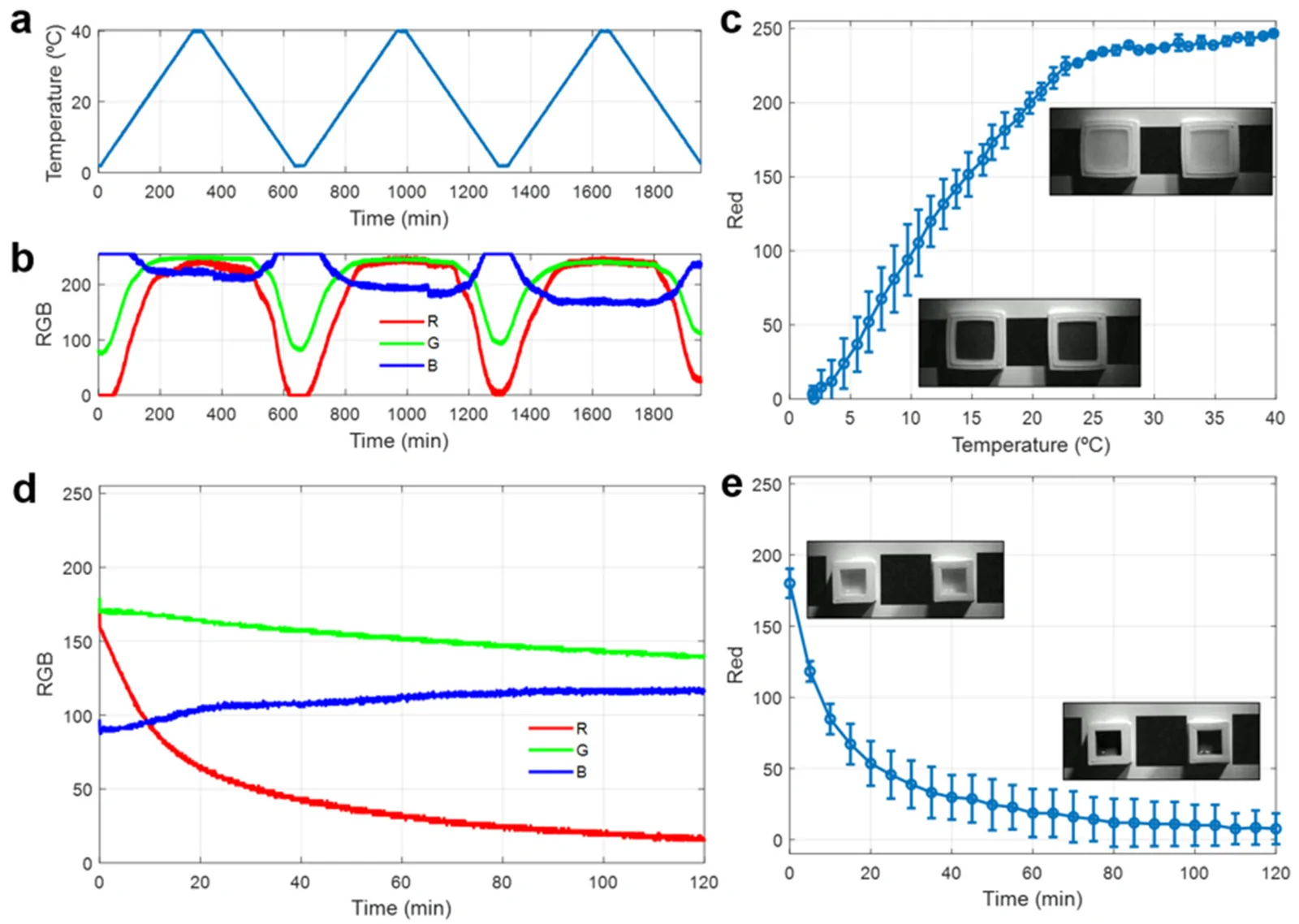
(**a**) Temperature profile applied during cyclic heating–cooling experiments. (**b**) Temporal evolution of the corrected RGB values for one of the sensors during the temperature cycles. (**c**) Red channel intensity as a function of temperature averaged for both sensors and over multiple cycles. Insets show representative grayscale images of the sensor in the blue (T < T_TH_) and colorless (T > T_TH_) states. (**d**) Temporal evolution of the RGB intensity values after exposure of a previously vacuum-sealed and UV-activated sensor to the ambient atmosphere. (**e**) Red channel intensity as a function of time after exposure to ambient atmosphere, averaged for two sensors. Insets show representative grayscale images of the sensor in the initial colorless (oxygen-free) state and the final blue (oxygen-exposed) state.

The colorimetric response of two identical temperature sensors subject to three consecutive heating–cooling cycles is shown in Figure 3. The applied cyclic temperature protocol (Figure 3a) was designed to evaluate the reversibility and stability of the thermochromic response across multiple transitions, while the use of two independent sensors allows assessment of sensor-to-sensor variability.

Figure 3b presents the corresponding temporal evolution of the RGB color channels extracted from the sensor images, after applying the W/B correction of Equation (1) to each image using black and white references printed next to the sensor to compensate for variations in illumination. In agreement with the UV–Vis spectroscopy results, a pronounced modulation of the red channel intensity is observed, closely following the applied temperature profile throughout the heating and cooling cycle. When the temperature decreases below the thermochromic transition temperature (T_TH_ = 13 °C), the sensor develops a blue coloration, resulting in strong attenuation of red wavelengths and a sharp decrease in the red channel intensity. Conversely, upon heating above T_TH_, the pigment transitions to its colorless state, leading to a substantial increase in red intensity. The visible color change in the sensors is shown in Figure S2a–d, while time-lapse videos of the transition are provided in the Supplementary Material as Video S1 (for the original RGB video) and Video S2 (grayscale video obtained from the red channel).

Both green and blue channels exhibit smaller intensity variations, the red channel being the one that provides the highest sensitivity to the thermochromic transition. In this sense, Figure 3c presents the red-channel intensity as a function of temperature, averaged for both sensors over all heating–cooling cycles. As observed, its response follows a linear increase with respect to the temperature followed by a plateau corresponding to the transparent state of the sensor. Regarding thermal hysteresis, the low standard deviation in Figure 3c, which includes both heating and cooling paths, indicates that the difference between both paths is small, with a maximum deviation of ~20 red-channel units (equivalent to ~2 °C) near the transition temperature. This is in agreement with our previous work on PDMS/thermochromic composites [18], where hysteresis was also found to be smaller than cycle-to-cycle variability. This standard deviation across cycles and devices demonstrates the reproducibility, robustness, and reversibility of the thermoresponsive system. According to these results, the red-channel signal can therefore be used as a single-channel grayscale input, as illustrated by the inset images in Figure 3c, which show the clear visual contrast between both colored (blue) and colorless states. In addition, the dynamic response of the temperature sensors after being transferred from a refrigerator (4 °C) to ambient temperature (27 °C), represented in Figure S3, shows t_50%_ and t_90%_ values (times required to reach 50% and 90% of the total red-channel intensity change) of 2.2 and 5 min, respectively, which demonstrates their capability for the fast detection of cold-chain breaks.

This strong binary contrast is particularly useful for subsequent threshold-based image processing and confirms the suitability of the sensor for integration into QR codes. For instance, in this scenario, applying a fixed mid-scale grayscale threshold (0.5, 127/255) to the corrected red-channel values would classify the sensor as “black” below 12.13 °C and as “white” above this temperature.

#### 3.4.2. Oxygen Responsive Sensor

The colorimetric response of the oxygen-responsive hydrogel sensor upon exposure to ambient atmosphere is shown in Figure 3d,e. Prior to the experiment, the sensors were vacuum-sealed and activated by UV irradiation as described in the Section 2, resulting in the photoreduction of methylene blue to its colorless leuco form. Immediately after unsealing, the sensors were placed under ambient conditions, and their color evolution was continuously monitored.

Figure 3d shows the temporal evolution of the RGB color channels extracted from the sensor images. Upon exposure to oxygen, a pronounced decrease in the red channel intensity is observed, accompanied by significantly smaller variations in the green and blue channels. This behavior is consistent with the oxidative recovery of methylene blue, whereby the colorless leuco form is re-oxidized to its blue state, leading to strong absorption in the red region of the visible spectrum, and is also in agreement with the UV–Vis spectroscopy results.

Again, the red channel exhibits the highest sensitivity to the colorimetric transition. Accordingly, Figure 3e presents the red-channel intensity as a function of time, averaged over two independent sensors. Thus, the response shows a steep initial decrease followed by a gradual approach to a stable plateau, corresponding to the fully oxidized, blue state of the sensor. Quantitatively, the t_50%_ and t_90%_ values after unsealing were 8.3 and 50 min, respectively, which enables the detection of oxygen ingress within minutes. In addition, the standard deviation throughout the experiment indicates good reproducibility of the oxygen-sensing response between sensors. Moreover, the inset grayscale images included in Figure 3e illustrate the clear visual contrast between the initial colorless state and the final blue state, thereby demonstrating the suitability of the sensor for straightforward threshold-based processing as well. The visible color change in the sensors is shown in Figure S2e–h, while time-lapse videos of the transition are also provided in the Supplementary Material as Video S3 (original RGB video) and Video S4 (grayscale video obtained from the red channel).

In order to verify that the oxygen sensor response is not affected by temperature, two vacuum-sealed sensors, one UV-activated (colorless) and one non-activated (blue), were subjected to a heating–cooling cycle between 4 and 35 °C while their color was continuously recorded (Figure S4, Videos S5 and S6). In both cases, the red-channel variation remained below ~13 units (5% of full scale) over the whole temperature range, which is negligible compared with the red-channel change associated with oxygen exposure (~170 units).

#### 3.4.3. Integration of Thermoresponsive and Oxygen Sensors into QR Codes

To demonstrate the simultaneous integration of temperature and oxygen sensing while preserving reliable access to digital information, both sensors were incorporated into a modified QR-code architecture designed to ensure permanent readability of the encoded data. Therefore, this strategy allows the QR code to consistently preserve essential information (such as product identification, batch or lot number, or shipping metadata) while concurrently monitoring the environmental state through embedded colorimetric sensors.

**Figure 4:**
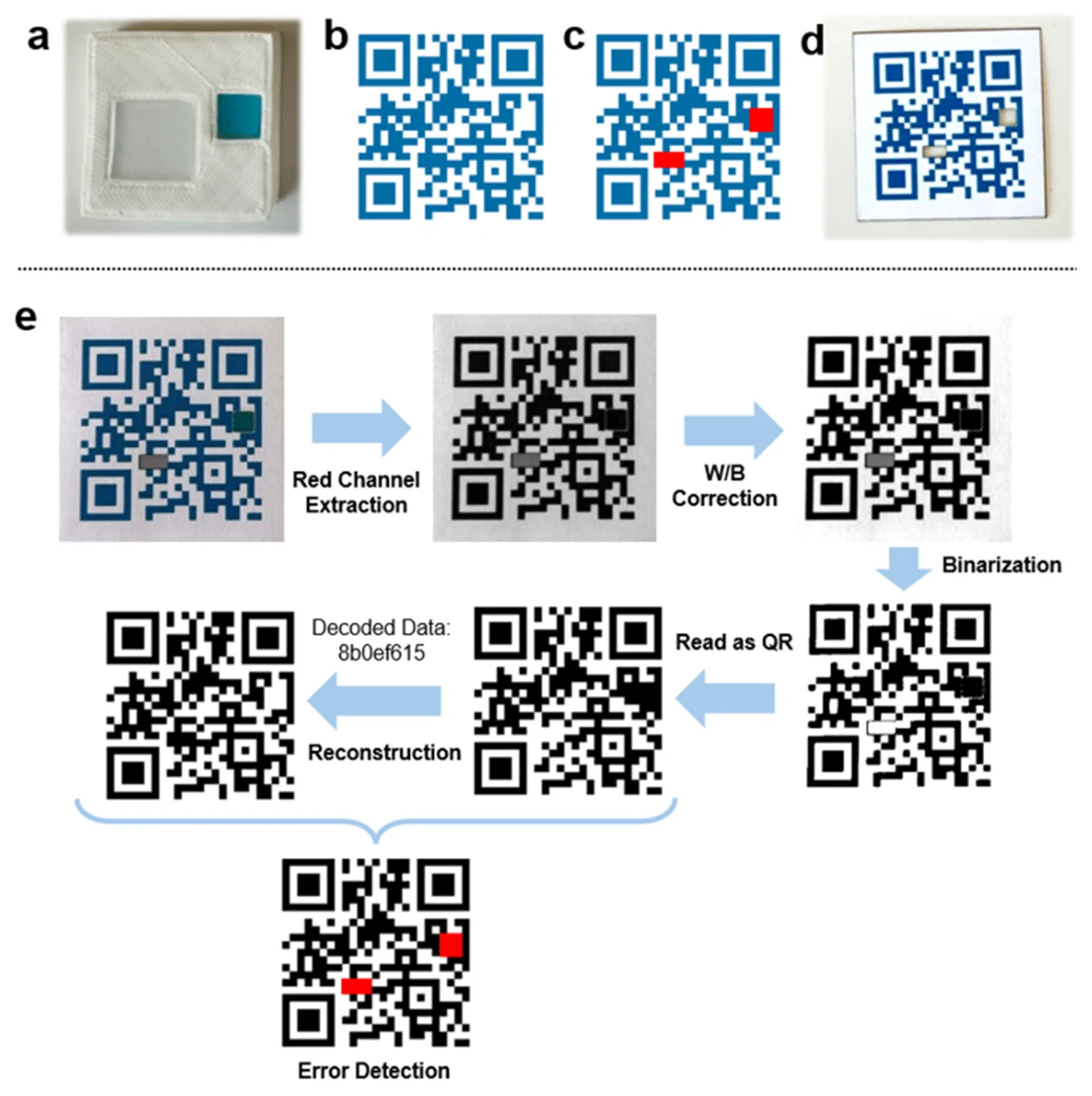
(**a**) 3D mold (40 × 40 mm) used and deposited colorimetric sensors. (**b**) Original QR code containing the UID: 8b0ef615. (**c**) Modules selected (in red) to be replaced by the colorimetric sensors. (**d**) Actual printed QR code after the laser cutting of the red regions of (**c**). (**e**) Algorithm for sensor status identification based on the error correction capability of QR codes.

For that, custom 3D-printed PLA molds were developed to deposit both sensing elements at fixed and predefined positions, as shown in Figure 4a. The overall dimensions of the mold were 40 × 40 × 10 mm, while the individual sensor cavity dimensions matched those described in the previous sections. The QR code was generated using the *segno* Python-based QR-code generator [63], which allows precise control over the encoded data, symbol version, and error-correction parameters. In this demonstration, the QR code encodes a 32-bit unique identifier (UID), which serves as a compact digital reference that can be directly associated with product-specific information such as batch number, shipment ID, or logistics metadata stored in an external database. The resulting QR code corresponds to Version 2, which provides a symbol size of 25 × 25 modules, offering a suitable balance between physical size, information capacity, and spatial flexibility for sensor integration [64]. An error-correction level H (high, ~30% redundancy) was selected to ensure robust decoding despite the intentional modification of selected data modules by the embedded colorimetric sensors, since this high level of redundancy enables the QR code to remain fully readable even when localized optical changes occur within the sensing regions. A Version 2-H symbol comprises 44 codewords (8-bit units), 28 of which provide Reed–Solomon error correction. Since the location of a corrupted codeword is unknown a priori, each erroneous codeword consumes two units of redundancy, so the code can correct up to 28/2 = 14 such codewords. Note that a codeword’s 8 bits are not a contiguous block of modules since the QR placement algorithm interleaves them in a zig-zag pattern across the symbol, so a compact group of modules typically spans several codewords.

The QR codes are printed on standard office paper in a bluish color with red channel set to zero (e.g., #006EA6). An example of QR code is provided in Figure 4b, which contains the UID: 8b0ef615. From the reference QR code, two non-critical data modules were identified and designated as sensing regions. In the intended application, the monitored product is supposed to be shipped under refrigerated and vacuum-sealed conditions, so the oxygen sensor must remain in its colorless (white) state due to oxygen absence, while the temperature sensor must appear blue when the storage temperature remains below the threshold. On this basis, one of the blue regions is replaced by the temperature-response sensor, whereas the oxygen-responsive sensor replaces a white region. As depicted in Figure 4c, in this example the selected regions cover 8 blue modules and 9 white modules, respectively. Each sensing region thus consists of a multi-module block (2 × 4 modules for the temperature sensor and 3 × 3 modules for the oxygen sensor). Given the printed module size of 1.2 mm (30 × 30 mm for the 25 × 25-module symbol), these windows measure approximately 4.8 × 2.4 mm and 3.6 × 3.6 mm, respectively, and are therefore smaller than the sensor cavities of the mold (17 × 17 mm and 10 × 10 mm). This ensures that the whole window area is covered by the sensing material despite small alignment tolerances between the printed label and the mold. The selected regions of the QR to be replaced by the colorimetric sensors are then removed by laser cutting from the printed QR code (Figure 4d). Notably, owing to the high error-correction capability of the QR code, the partial substitution of data modules by colorimetric sensing elements do not prevent successful decoding even when the sensors change their optical state. In particular, these 17 modules intersect 7 codewords, which correspond to half the symbol’s correction capacity, thus leaving 7 codewords (~16%) to tolerate further damage (e.g., scratches, stains). More generally, for a symbol with *x* correctable codewords, safe integration requires the sensing regions to occupy no more than *x*/2 codewords, preserving at least half of the correction capacity for unintended damage [65]. In practice, we would recommend sizing each sensing region between 6 and 9 modules, with a minimum side of 2 modules, which ensures adequate visualization of the colorimetric response (avoiding elongated, 1-module-wide strips) while keeping codeword usage low. Across 300 randomly generated shipments in our implementation, regions constrained to this 6–9 module range intersected 3–8 codewords combined, with only 0.7% of instances exceeding the *x*/2 margin. In every case the maximum codeword usage observed remained below the hard limit of 14.

On the other side, Figure 4e illustrates the image-processing workflow used to extract sensor information while preserving QR readability. First, as done for the color characterization of the sensors, the QR image is converted to the red channel only. Afterward, a W/B correction is applied to enhance contrast, as detailed in Equation (1) of the Section 2. For that, a percentile-based contrast normalization approach is employed [66], in which the black and white reference levels are estimated from the 20th and 80th intensity percentiles, respectively. In this case, no additional calibration elements are required, since the printed QR modules themselves, whose colors are known, serve as internal “black” and “white” references. Subsequently, the red-channel grayscale image was converted into a binary image through a thresholding operation [41] using a fixed threshold value of 0.25 (65/255) to classify pixels into black and white according to Equation (2). This threshold was selected on the basis of the distribution of normalized gray levels of the printed QR modules and of the sensing modules in both states (Figure S5). After normalization, the colored states of both sensors remain close to the printed dark modules, whereas their colorless states exhibit intermediate gray levels (77–134) owing to the translucency of the sensing materials and the shadows of the laser-cut windows. Consequently, the threshold was placed below mid-scale, within the gap between both sensor states, while the printed QR modules remain correctly classified. Given that the black and white references are computed for each image from the QR code itself, which contains a large and approximately balanced number of dark and light printed modules, the normalization acts as an internal calibration that compensates for global variations in illumination intensity, color temperature, exposure and white balance, as well as for possible differences in image acquisition between smartphone cameras. Nevertheless, a systematic validation with a wider range of acquisition conditions, such as strong nonuniform illumination, is planned as future work.

Once the sensor-integrated QR image is binarized, it is processed using the standard QR decoding functions available in OpenCV. It should be noted that, regardless of the state of the embedded sensors or the presence of other local defects within the QR code, the inherent error-correction capability of the QR standard allows reliable decoding of the encoded content, in this case the unique identifier (UID). Furthermore, since each decoded UID is uniquely associated with a specific QR pattern, the original error-free QR code can be reconstructed from the decoded information. Subsequently, the experimentally acquired QR code and the reconstructed reference QR code are compared on a module-by-module basis, enabling the identification of deviations between both patterns. For each QR identifier, a database can store not only the associated product information, such as product identification, batch number, lot number, or shipping metadata, but also the location and type of the sensing modules embedded within the QR structure. In the implementation presented here, the oxygen sensor is associated with modules [[22 11],[24 13]], while the temperature sensor corresponds to modules [[9 17],[12 18]], where each pair indicates the (x, y) = (column, row) indices of the top-left and bottom-right modules of the sensing region, starting from 1 at the top-left module of the symbol, with columns increasing to the right and rows increasing downwards. It should be noted that several candidate sensing regions fulfill the conditions described above (each region comprising between 6 and 9 modules, with a minimum side of 2 modules and no overlapping or touching). In our implementation, after identifying all valid candidates, the final layout is selected randomly among them, being one set over dark (blue) modules for the temperature sensor, and another over light (white) modules for the oxygen sensor. In this scenario, the database must store the position of the sensors, which requires the reading device either to have real-time internet connectivity to query the database or to periodically store a local copy of it. This only requires a few additional bytes per item (sensor type and module coordinates) and can therefore be handled by standard traceability databases in high-volume supply chains. The internet connectivity constraint could be overcome by computing the layout locally through a key-based derivation. Instead of selecting the sensing regions randomly, the selection can be driven by a seed computed from a secret key and the UID (e.g., using Hash-based Message Authentication Code (HMAC) [67]) for instance by choosing the candidate whose index equals the seed modulo the number of valid options. In this way, a reading application holding the key can reproduce the layout offline, whereas the layout remains unpredictable without the key, thus preserving its authentication function.

Finally, the state of each sensor is determined by analyzing the modules assigned to the corresponding sensing region. Oxygen exposure and/or temperature excursions are identified when more than 50% of the modules associated with a given sensor differ from their expected state in the reconstructed QR pattern. The 50% criterion was selected to prevent isolated misclassified modules, caused by dust, partial scratches, local reflections or edge effects at the boundaries of the laser-cut windows, from triggering false alarms. This approach enables simultaneous retrieval of digital information and environmental-state monitoring within a single passive QR-based platform, while maintaining full QR readability throughout the sensing process.

Regarding the different kinetics of both sensors, it should be noted that in a real logistics scenario the package would be scanned at different checkpoints along the supply chain rather than continuously. Since both sensors respond within minutes, a sensor undergoing a transition during one scan would be correctly identified at the subsequent checkpoint, while the stored images and decoded states provide a traceable record for later review. Moreover, the oxygen sensor is irreversible, thus recording any oxygen ingress, whereas the thermochromic sensor reports the temperature at the time of scanning. However, irreversible thermochromic formulations could be used when a record of thermal history is required [68]. Therefore, note that the oxygen sensor integrated in this work is intended as a single-use indicator. It is activated once after packaging and, upon exposure to air, remains in its oxidized blue state, thus providing an irreversible record of oxygen ingress. As reported in the reference study [45], the initial color is not fully recovered after re-oxidation and repeated activation cycles lead to a progressive loss of performance due to partial photodegradation of MB, water loss and consumption of the sacrificial electron donor.

**Figure 5:**
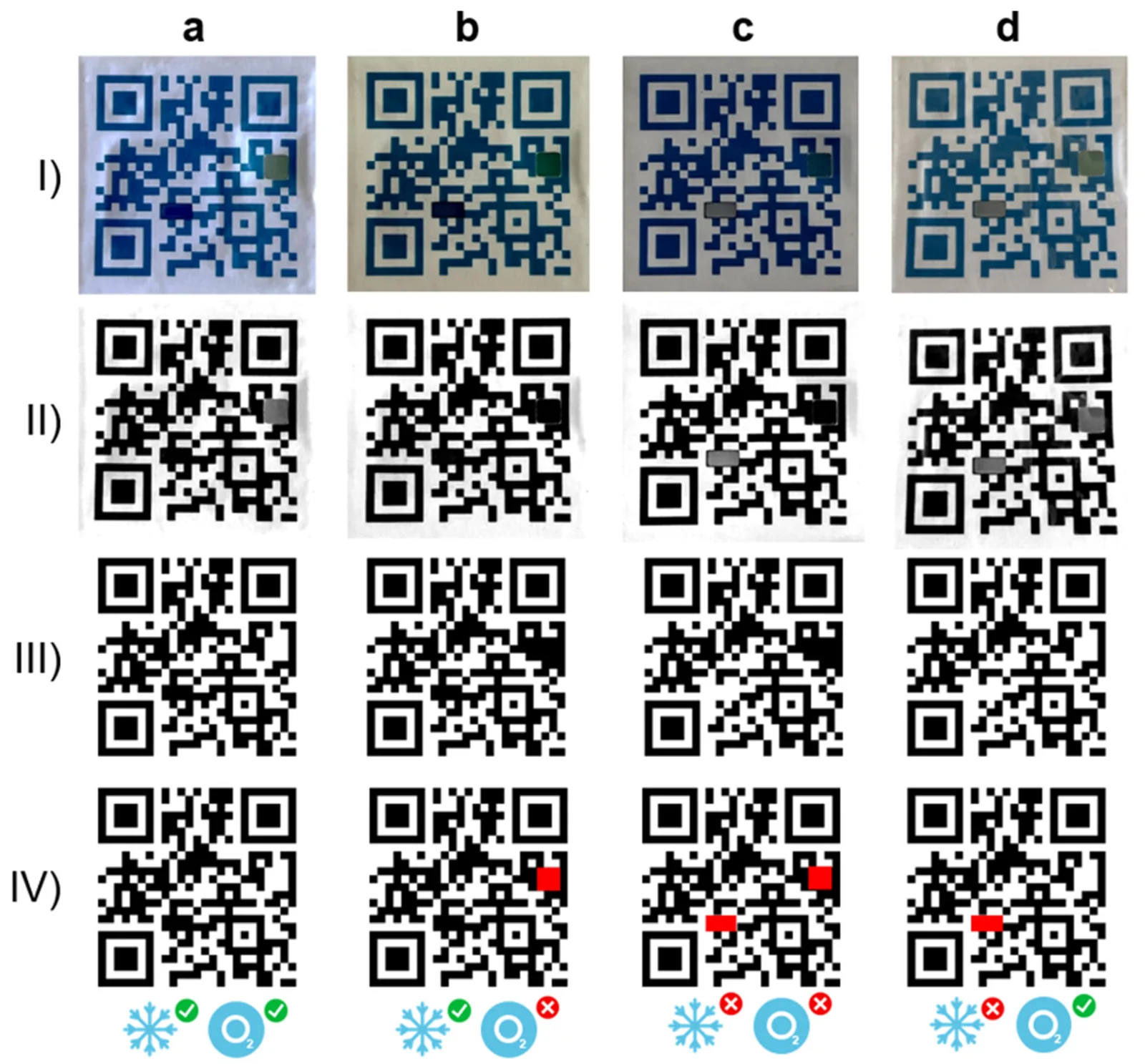
Full QR code sensor under the different conditions: (**a**) refrigerated and vacuum-sealed, (**b**) refrigerated and non-sealed, (**c**) non-refrigerated and non-sealed, and (**d**) non-refrigerated and vacuum-sealed. Different images correspond to (I) raw image under (no controlled light conditions), (II) W/B contrast-corrected image, (III) identified QR code, and (IV) module errors (in red) with respect to the expected QR code.

To demonstrate the feasibility of the proposed approach in a smart-food-packaging scenario, one of the sensor-integrated QR codes was vacuum-sealed inside a commercial food-grade plastic container together with an oxygen absorber commonly used in food preservation, as shown in Figure S6. The sensing module was then evaluated under the four possible storage conditions illustrated in Figure 5: (a) refrigerated and vacuum-sealed, (b) refrigerated and non-sealed, (c) non-refrigerated and non-sealed, and (d) non-refrigerated and vacuum-sealed. For the refrigerated conditions, the package was stored in a refrigerator at 4 °C for 24 h, whereas the non-refrigerated conditions correspond to storage at room temperature (~25 °C) for 1 h. Non-sealed conditions were obtained by opening the package and exposing it to ambient air for 2 h before image acquisition. In addition, to assess the performance of the proposed methodology under realistic operating conditions, images were acquired using a standard smartphone (Xiaomi Redmi Note 10 Pro) without controlled illumination or uniform lighting. The raw images corresponding to each scenario are shown in step (I) of Figure 5. As seen, the four images were taken under different lighting conditions, resulting in different black and white reference levels estimated by the percentile-based normalization, which range from 0 to 15 for *R*_B_ and from 148 to 171 for *R*_W_, respectively. The rest of panels in Figure 5 illustrate the successive stages of the recognition algorithm: (II) W/B contrast correction, (III) QR identification and decoding, and (IV) module-level error detection. Despite the variability in illumination conditions, the QR code was successfully decoded in all cases due to its intrinsic error-correction capability. Furthermore, all detected errors were exclusively associated with the sensing regions, leading to a correct identification of the four environmental states. In all cases shown, 100% of the modified modules corresponding to the activated sensing elements were correctly detected.

Note that from the QR code encoding its UID, from all candidate rectangular regions that satisfy the sensing-area requirements, two are selected randomly. Because the position of the sensing modules is therefore not fixed and is tied to the encoded identifier, this provides an additional layer of tamper resistance and product authentication, making unauthorized replication or manipulation of sensor-enabled QR labels considerably more difficult.

These results demonstrate that the proposed strategy enables simultaneous digital information retrieval and environmental-state monitoring within a single passive QR-based platform while preserving full QR readability. As seen, unlike conventional QR-based sensing approaches that rely on code degradation or loss of readability, the proposed methodology decouples sensing functionality from data decoding and can therefore be readily integrated into existing QR-processing pipelines. In addition, the platform is inherently scalable, as the sensing modules can be associated with any colorimetric sensor and distributed across one or multiple QR modules to increase robustness and support the simultaneous detection of several analytes.

## 4. Conclusions

This work presents a QR-code-integrated sensing platform capable of simultaneously monitoring temperature and oxygen while preserving the information-storage and decoding functions of conventional QR codes. A thermochromic PDMS composite and a photoactivatable ZnO-alginate hydrogel were successfully developed and characterized, confirming their suitability as colorimetric sensing elements. Spectroscopic analysis revealed that both sensors exhibit their dominant optical response in the red region of the visible spectrum (610–660 nm), enabling a sensitive and robust single-channel image-processing strategy based on red-channel grayscale analysis. The thermochromic sensor exhibits a transition threshold of 13 °C with a red-channel change of ~245 units (out of 255) between its colored and colorless states with t_50%_ and t_90%_ values of 2.2 and 5 min after removal from refrigeration; while the oxygen sensor shows a red-channel change of ~170 units, with t_50%_ and t_90%_ values of 8.3 and 50 min after exposure to air.

By replacing selected non-critical QR modules with sensing elements and exploiting the intrinsic error-correction capability of QR codes, environmental monitoring was achieved without compromising QR readability. The proposed image-processing workflow, based on red-channel extraction, contrast correction, binarization, QR decoding, and module-level comparison with the expected QR pattern, enabled reliable determination of sensor states directly from smartphone-acquired images.

The platform successfully identified the four possible combinations of refrigeration and vacuum-sealing conditions in a smart-food-packaging demonstration performed under uncontrolled illumination, highlighting its robustness under realistic operating conditions. In addition to preserving full decoding functionality, the proposed architecture offers considerable design flexibility, since the number, position, and distribution of sensing modules can be adapted without affecting QR performance. This feature not only facilitates the integration of additional analytes and multi-sensor configurations but also introduces an additional degree of tamper resistance and product authentication through configurable sensor layouts linked to the encoded identifier.

Overall, the proposed strategy transforms QR codes from passive data carriers into multifunctional platforms capable of simultaneously providing digital traceability and environmental monitoring. The approach offers a low-cost, passive, and scalable solution with significant potential for smart packaging, cold-chain monitoring, pharmaceutical logistics, and other applications requiring combined product identification and condition tracking. In this sense, future work will focus on evaluating the performance of the platform under condensation during cold-chain transfers and mechanical stress during transport, and on validating it with a larger number of sensors from several fabrication batches.

## Figures and Tables

**Table 1:** Examples of colorimetric sensor-based QR codes reported in the literature.

Sensor	Materials	Static + Dynamic Information	Contrast Correction	Refs.
Temperature and O_2_	Alginate-based hydrogels embedded with ZnO + Thermochromic pigments in PDMS	Yes	Yes	This work
Temperature	Lanthanide-doped luminescent nanoparticles	No	No	[22,23]
	Polydiacetylene precursors, phosphorus-doped carbon dots in PVA, and microencapsulated thermochromic pigments deposited via inkjet/screen printing	No	No	[24,25,26]
	Polyacrylic acid-reinforced gelatin (PAA/gelatin), hydroxypropyl cellulose/sodium carboxymethyl cellulose (HPC/CMC) composites, and stretchable photochromic hydrogels	No	No	[27,28,29]
pH	Volatile basic nitrogen compounds (amines, NH_3_)	No	No	[30]
RH	Photonic crystal (PC) film with P(AM-MBA) nanogels and TiO_2_ nanoparticles	No	No	[31]
Escherichia coli	p-dimethylaminocinnamaldehyde in paper microfluidic platform	No	No	[32]
NH_3_, CH_2_O and H_2_S	Flexible printed substrate with immobilized colorimetric indicators (pH dye, primary amine, Cu(II)–PAN complex)	Yes	Yes	[34]
Temperature, RH, CO_2_	Thermochromic ink; crystal violet/Nafion humidity-sensitive coating; α-naphtholphthalein/tetraoctylammonium hydroxide (TOAOH) CO_2_-sensitive indicator	Yes	Yes	[8]

## Data Availability

The original contributions presented in this study are included in the article/Supplementary Material. Further inquiries can be directed to the corresponding author.

## Supplementary Materials

The following supporting information can be downloaded at: https://www.mdpi.com/article/10.3390/s26206496/s1. Figure S1. Setup used for the UV activation of the vacuum-sealed oxygen sensor: UV LED lamp (Eleganted, ELE-PG365-100W, λ = 365 nm, 100 W) placed approximately 15 cm above the package, with an exposure time of 90 min at room temperature. Figure S2. Representative images of the temperature and oxygen sensors in their different sensing states and the corresponding red-channel grayscale images. (a) Temperature sensor in its colored state (T < T_TH_) and (b) its red-channel grayscale representation. (c) Temperature sensor in its colorless state (T > T_TH_) and (d) its red-channel grayscale representation. (e) Photoactivated oxygen sensor immediately after exposure to ambient atmospheric conditions and (f) its red-channel grayscale representation. (g) Oxygen sensor after 60 min of exposure to ambient atmospheric conditions and (h) its red-channel grayscale representation. Figure S3. Red-channel intensity response of the temperature sensor (averaged for two sensors) after being transferred from a refrigerator (4 °C) to ambient temperature (27 °C). Figure S4. (a) Temperature profile applied to two vacuum-sealed oxygen sensors. (b) Relative variation of the red channel (ΔR, % of full scale) of a UV-activated (colorless) and a non-activated (blue) sensor during the heating–cooling cycle. Figure S5. (a) Histogram of the normalized red-channel gray levels of the printed dark and light QR modules and of the sensing modules in their colored and colorless states, extracted from the four images of Figure 5 (ii). (b) Mean gray level of each module where the solid line indicates the binarization threshold (65/255). Figure S6. Vacuum-packed sensor module. Video S1. Color change of the temperature sensor in RGB. Video S2. Color change of the temperature sensor in grayscale from red channel. Video S3. Color change of the oxygen sensor in RGB. Video S4. Color change of the oxygen sensor in grayscale from red channel. Video S5. Activated and non-activated oxygen sensors during a heating–cooling cycle between 4 and 35 °C in RGB. Video S6. Activated and non-activated oxygen sensors during a heating–cooling cycle between 4 and 35 °C in grayscale from red channel.

## References

[1] Hernández-Neuta I., Neumann F., Brightmeyer J., Ba Tis T., Madaboosi N., Wei Q., Ozcan A., Nilsson M. (2019). Smartphone-based Clinical Diagnostics: Towards Democratization of Evidence-based Health Care. J. Intern. Med..

[2] Ozcan A. (2014). Mobile Phones Democratize and Cultivate Next-Generation Imaging, Diagnostics and Measurement Tools. Lab Chip.

[3] Wyrwa J., Barska A. (2017). Innovations in the Food Packaging Market: Active Packaging. Eur. Food Res. Technol..

[4] Herrmann M., Boehme P., Mondritzki T., Ehlers J.P., Kavadias S., Truebel H. (2018). Digital Transformation and Disruption of the Health Care Sector: Internet-Based Observational Study. J. Med. Internet Res..

[5] Jayaraman P.P., Forkan A.R.M., Morshed A., Haghighi P.D., Kang Y. (2020). Healthcare 4.0: A Review of Frontiers in Digital Health. WIREs Data Min. Knowl. Discov..

[6] Villegas-Ch W., García-Ortiz J., Sánchez-Viteri S. (2024). Toward Intelligent Monitoring in IoT: AI Applications for Real-Time Analysis and Prediction. IEEE Access.

[7] Chang J.H. (2014). An Introduction to Using QR Codes in Scholarly Journals. Sci. Ed..

[8] Escobedo P., Ramos-Lorente C.E., Ejaz A., Erenas M.M., Martínez-Olmos A., Carvajal M.A., García-Núñez C., de Orbe-Payá I., Capitán-Vallvey L.F., Palma A.J. (2023). QRsens: Dual-Purpose Quick Response Code with Built-in Colorimetric Sensors. Sens. Actuators B Chem..

[9] Fernandez-Carames T.M., Fraga-Lamas P. (2018). A Review on Human-Centered IoT-Connected Smart Labels for the Industry 4.0. IEEE Access.

[10] Raman R., Kumar V., Pillai B.G., Rabadiya D., Divekar R., Vachharajani H. Implementing QR Code-Enabled Smart Documents: A Fusion of Distributed Databases and Digital Signatures. Proceedings of the 2024 Second International Conference on Data Science and Information System (ICDSIS), Hassan, India, 17–18 May 2024.

[11] Khatri R., Jadhav R., Gherwada D., Kotak V., Malvankar R., Nadkarni S. A Tiered Patient-Centric Emergency Medical Access System Using Dynamic QR-Codes. Proceedings of the 2026 7th International Conference on Intelligent Communication Technologies and Virtual Mobile Networks (ICICV), Tirunelveli, India, 20–22 May 2026.

[12] Almousa H., Alsuhibany S.A. (2026). QR Code Security: An Adaptive Retraining Approach for Dynamic URL-Based Threat Detection. Sci. Rep..

[13] Hasan M.W. (2023). Building an IoT Temperature and Humidity Forecasting Model Based on Long Short-Term Memory (LSTM) with Improved Whale Optimization Algorithm. Mem.-Mater. Devices Circuits Syst..

[14] Fernández-Ramos M.D., Pageo-Cabrera M., Capitán-Vallvey L.F., Pérez de Vargas-Sansalvador I.M. (2021). Inkjet-Printed O_2_ Gas Sensors in Intelligent Packaging. Analyst.

[15] Zhang X., Chen Z., Zuo X. (2020). Chloroauric Acid/Silver Nanoparticle Colorimetric Sensors for Antioxidant Discrimination Based on a Honeycomb Ag-Au Nanostructure. ACS Sustain. Chem. Eng..

[16] Li W., Cai T., Zhu W., Tian J., Liu Y., Chen H., Peng H. (2026). A Smartphone–Assisted Colorimetric Sensor Array Based on Trimetallic Nanozyme for High–Throughput and on–Site Discrimination of Antioxidants in Vegetables and Teas. Talanta.

[17] Jalal U.M., Jin G.J., Shim J.S. (2017). Paper–Plastic Hybrid Microfluidic Device for Smartphone-Based Colorimetric Analysis of Urine. Anal. Chem..

[18] López-León C., Salcedo-Abraira P., Moyano C., Toral V., Morales D.P., Salinas-Castillo A., Romero F.J. (2026). Smart Colorimetric Temperature Sensor with Thermoresponsive Polymers and Automatic Image-Based Identification. Sens. Actuators A Phys..

[19] El Matbouly H., Escobedo P., Dahiya R. Flexible Metasurface QR Code for Simultaneous Identification and Sensing. Proceedings of the 2021 IEEE International Conference on Flexible and Printable Sensors and Systems (FLEPS), Manchester, UK, 20–23 June 2021.

[20] Ramalho J.F.C.B., António L.C.F., Correia S.F.H., Fu L.S., Pinho A.S., Brites C.D.S., Carlos L.D., André P.S., Ferreira R.A.S. (2018). [INVITED] Luminescent QR Codes for Smart Labelling and Sensing. Opt. Laser Technol..

[21] Zhang Y., Li Y., Chen K., Ma Z., Wang X., Shi L., Ran R. (2019). Body Temperature Triggered Customized Patterned Thermo-Sensitive Hydrogel for Information Storage and Display. Polymer.

[22] Ramalho J.F.C.B., Correia S.F.H., Fu L., António L.L.F., Brites C.D.S., André P.S., Ferreira R.A.S., Carlos L.D. (2019). Luminescence Thermometry on the Route of the Mobile-Based Internet of Things (IoT): How Smart QR Codes Make It Real. Adv. Sci..

[23] Lara Lúcia Fernandes A. (2018). Códigos QR Luminescentes Multiplexados Para Etiquetas Inteligentes. Master’s Thesis.

[24] Li Z., Li Y., Zhou M., Zhu X., Wang R., Zhang Y.-F., Sun W., Li H., Zhang H., Li P. (2025). 3D Printing of Thermochromic Multilayer Flexible Film for Multilevel Information Encryption. J. Colloid Interface Sci..

[25] Bao X., Zhu X., Tian Z., Wang H., Li H., Yuan X. (2025). Dual-Mode Thermochromic Afterglow in Phosphorus-Doped Carbon Dot Composites for Visible Light-Activated Information Encryption. J. Colloid Interface Sci..

[26] Yoon B., Shin H., Kang E.-M., Cho D.W., Shin K., Chung H., Lee C.W., Kim J.-M. (2013). Inkjet-Compatible Single-Component Polydiacetylene Precursors for Thermochromic Paper Sensors. ACS Appl. Mater. Interfaces.

[27] Luo S., Hu C.-Y., Huang S., Xu X. (2025). Polyacrylic Acid-Reinforced Gelatin Hydrogels with Enhanced Mechanical Properties, Temperature-Responsiveness and Antimicrobial Activity for Smart Encryption and Salmon Freshness Monitoring. J. Colloid Interface Sci..

[28] Yi Z., Song Z., Wu M., He M. (2025). Dual Temperature-Sensitive Hydroxypropyl Cellulose/Sodium Carboxymethyl Cellulose (HPC/CMC) Composite Hydrogel for Smart Window, Information Encryption and Temperature Monitoring. Polymer.

[29] Pan Z., Li Y., Ren R., Luo Y., Zhang S., Dac Le V.-H., Giang Phan V.H., He Y., Li Y. (2026). Highly Transparent, Stretchable, and 3D-Printable Photochromic Hydrogel for Information Encryption and Coding. Polymer.

[30] Xu Y., Liu Z., Liu R., Luo M., Wang Q., Cao L., Ye S. (2021). Inkjet-Printed PH-Sensitive QR Code Labels for Real-Time Food Freshness Monitoring. J. Mater. Sci..

[31] Kou D., Ma W., Zhang S., Lutkenhaus J.L., Tang B. (2018). High-Performance and Multifunctional Colorimetric Humidity Sensors Based on Mesoporous Photonic Crystals and Nanogels. ACS Appl. Mater. Interfaces.

[32] Burklund A., Saturley-Hall H.K., Franchina F.A., Hill J.E., Zhang J.X.J. (2019). Printable QR Code Paper Microfluidic Colorimetric Assay for Screening Volatile Biomarkers. Biosens. Bioelectron..

[33] Kim H., Seo M., Kim J., Kwon D., Choi S., Kim J.W., Myoung J. (2019). Highly Stretchable and Wearable Thermotherapy Pad with Micropatterned Thermochromic Display Based on Ag Nanowire–Single-Walled Carbon Nanotube Composite. Adv. Funct. Mater..

[34] Engel L., Benito-Altamirano I., Tarantik K.R., Pannek C., Dold M., Prades J.D., Wöllenstein J. (2021). Printed Sensor Labels for Colorimetric Detection of Ammonia, Formaldehyde and Hydrogen Sulfide from the Ambient Air. Sens. Actuators B Chem..

[35] Cao R., Lou Y., Yang Z., Sun L., Nilghaz A., Li M., Tian J. (2025). Stacking Paper Sheets into Multi-Purpose Quick Response Sensing Code with Built-in Nitrocellulose-Membrane-Free Lateral Flow Assay for Detecting Tetracycline in Food Samples. Food Chem..

[36] Benito-Altamirano I., Crugeira F., Marchena M., Prades J.D. (2024). The Application of Back-Compatible Color QR Codes to Colorimetric Sensors. Proceedings.

[37] Esteves M., Tirapu-Azpiroz J., Lopes Marcondes Marçal D.V., Ferreira Silva A., Ohta R., Steiner M., Coté G.L. Illumination Compensation Algorithm for Colorimetric Detection of Microfluidic Paper-Based Devices with a Smartphone. Proceedings of the Optical Diagnostics and Sensing XXI: Toward Point-of-Care Diagnostics.

[38] Marín-Sánchez A., Ortiz-Gómez I., Gallego-Méndez D., Rodríguez-Domínguez C., Fermín Capitán-Vallvey L., Morales D.P., Antonio Álvarez-Bermejo J., Castillo E., Salinas-Castillo A. (2022). Smart Quick Response Code for Multianalyte Determination. Measurement.

[39] Hariram B., Karthika R.N., Anandasayanam K., Maheswara Pandian G. (2023). Dynamic E-Authentication Attendance System Using QR Code and OTP. Proceedings of the International Conference on Emerging Trends in Expert Applications & Security.

[40] Kim S.D., Koo Y., Yun Y. (2017). A Smartphone-Based Automatic Measurement Method for Colorimetric PH Detection Using a Color Adaptation Algorithm. Sensors.

[41] Benito-Altamirano I., Martínez-Carpena D., Casals O., Fábrega C., Waag A., Prades J.D. (2023). Back-Compatible Color QR Codes for Colorimetric Applications. Pattern Recognit..

[42] Vu C.H.T., Won K. (2013). Novel Water-Resistant UV-Activated Oxygen Indicator for Intelligent Food Packaging. Food Chem..

[43] Shin J., Han S.G., Lee W. (2012). Dually Tunable Inverse Opal Hydrogel Colorimetric Sensor with Fast and Reversible Color Changes. Sens. Actuators B Chem..

[44] Yi H., Lee S.-H., Kim D., Jeong H.E., Jeong C. (2022). Colorimetric Sensor Based on Hydroxypropyl Cellulose for Wide Temperature Sensing Range. Sensors.

[45] Sattayapanich K., Chaiwat W., Boonmark S., Bureekaew S., Sutthasupa S. (2022). Alginate-Based Hydrogels Embedded with ZnO Nanoparticles as Highly Responsive Colorimetric Oxygen Indicators. New J. Chem..

[46] Aparicio Ceja M.E., Carbajal Arizaga G.G. (2015). Utilidad de La Difracción de Rayos x En Las Nanociencias. Mundo Nano. Rev. Interdiscip. En Nanociencias Y Nanotecnología.

[47] Gupta N.S., Lee K.-S., Labouriau A. (2021). Tuning Thermal and Mechanical Properties of Polydimethylsiloxane with Carbon Fibers. Polymers.

[48] Maji D., Lahiri S.K., Das S. (2012). Study of Hydrophilicity and Stability of Chemically Modified PDMS Surface Using Piranha and KOH Solution. Surf. Interface Anal..

[49] Efimenko K., Wallace W.E., Genzer J. (2002). Surface Modification of Sylgard-184 Poly(Dimethyl Siloxane) Networks by Ultraviolet and Ultraviolet/Ozone Treatment. J. Colloid Interface Sci..

[50] Paisoonsin S., Pornsunthorntawee O., Rujiravanit R. (2013). Preparation and Characterization of ZnO-Deposited DBD Plasma-Treated PP Packaging Film with Antibacterial Activities. Appl. Surf. Sci..

[51] Motelica L., Ficai D., Oprea O., Ficai A., Trusca R.-D., Andronescu E., Holban A.M. (2021). Biodegradable Alginate Films with ZnO Nanoparticles and Citronella Essential Oil—A Novel Antimicrobial Structure. Pharmaceutics.

[52] Uddin M.T., Islam M.A., Mahmud S., Rukanuzzaman M. (2009). Adsorptive Removal of Methylene Blue by Tea Waste. J. Hazard. Mater..

[53] Tam S.K., Dusseault J., Polizu S., Ménard M., Hallé J.-P., Yahia L. (2005). Physicochemical Model of Alginate–Poly-l-Lysine Microcapsules Defined at the Micrometric/Nanometric Scale Using ATR-FTIR, XPS, and ToF-SIMS. Biomaterials.

[54] Ovchinnikov O.V., Evtukhova A.V., Kondratenko T.S., Smirnov M.S., Khokhlov V.Y., Erina O.V. (2016). Manifestation of intermolecular interactions in FTIR spectra of methylene blue molecules. Vib. Spectrosc..

[55] Janaki A.C., Sailatha E., Gunasekaran S. (2015). Synthesis, Characteristics and Antimicrobial Activity of ZnO Nanoparticles. Spectrochim. Acta A Mol. Biomol. Spectrosc..

[56] Toledo e Silva S.H., Bierhalz A.C.K., Moraes Â.M. (2025). Influence of Nanoparticle Content and Cross-Linking Degree on Functional Attributes of Calcium Alginate-ZnO Nanocomposite Wound Dressings. Membranes.

[57] Ma J., Sun Z., Wang Z., Zhou X. (2016). Preparation of ZnO–Cellulose Nanocomposites by Different Cellulose Solution Systems with a Colloid Mill. Cellulose.

[58] Trandafilović L.V., Božanić D.K., Dimitrijević-Branković S., Luyt A.S., Djoković V. (2012). Fabrication and Antibacterial Properties of ZnO–Alginate Nanocomposites. Carbohydr. Polym..

[59] Li X., Zhang X., Li L., Huang L., Zhang W., Ye J., Hong J. (2016). Preparation of Nano-ZnO/Regenerated Cellulose Composite Particles via Co-Gelation and Low-Temperature Hydrothermal Synthesis. Mater. Lett..

[60] Belashov A.V., Zhikhoreva A.A., Huang Z., Lin F., Semenova I.V., Vasyutinskii O.S., Qu J. (2026). Time-Resolved Fluorescence Imaging of Methylene Blue Reveals Heterogeneous Polarity in Living Cells. J. Photochem. Photobiol. B.

[61] Jacobs K.Y., Schoonheydt R.A. (2001). Time Dependence of the Spectra of Methylene Blue−Clay Mineral Suspensions. Langmuir.

[62] Fernández-Pérez A., Marbán G. (2020). Visible Light Spectrosc. Anal. Methylene Blue Water; What Comes After Dimer?. ACS Omega.

[63] Segno Developers Segno: Python QR Code and Micro QR Code Encoder Documentation. https://segno.readthedocs.io/en/latest/.

[64] Wu D.-C., Wu Y.-M. (2020). Covert Communication via the QR Code Image by a Data Hiding Technique Based on Module Shape Adjustments. IEEE Open J. Comput. Soc..

[65] Wicker S.B., Bhargava V.K. (1999). Reed-Solomon Codes and Their Applications.

[66] Zhang W., Liu Q., Feng Y., Cai L., Zhuang P. (2024). Underwater Image Enhancement via Principal Component Fusion of Foreground and Background. IEEE Trans. Circuits Syst. Video Technol..

[67] Krawczyk H. (2010). Cryptographic Extraction and Key Derivation: The HKDF Scheme. Annual Cryptology Conference.

[68] Galliani D., Mascheroni L., Sassi M., Turrisi R., Lorenzi R., Scaccabarozzi A., Stingelin N., Beverina L. (2015). Thermochromic Latent-Pigment-Based Time–Temperature Indicators for Perishable Goods. Adv. Opt. Mater..

