# Supplementary material for: QR-Code-Integrated Colorimetric Sensors for Simultaneous Temperature Threshold and Oxygen Detection
Source: Sensors (Basel). 2026 Oct 14;26(20):6496. doi: 10.3390/s26206496 (PMC13708874; doi:10.3390/s26206496)
Supplement: Supplementary file 1 [file sensors-26-06496-s001.zip › sensors-4580731-supplementary.pdf]

Supplementary Material

QR-Code-Integrated Colorimetric Sensors for Simultaneous Temperature Threshold and Oxygen Detection

Carmen López-León [1], César Moyano [2], Víctor Toral [3], Diego P. Morales [3], Alfonso Salinas-Castillo [1], Pablo Salcedo-Abraira [4] and Francisco J. Romero [3]*

[1] Department of Analytical Chemistry, Faculty of Sciences, University of Granada, Av. Fuente Nueva s/n, Granada 18071, Spain
[2] Spica BioTech SL, Calle Pintor Maldonado 23 (9), Granada 18007, Spain
[3] Department of Electronics and Computer Technology, Faculty of Sciences, University of Granada, Av. Fuente Nueva s/n, Granada 18071, Spain
[4] Department of Inorganic Chemistry, Faculty of Sciences, University of Granada, Av. Fuente Nueva s/n, Granada 18071, Spain
* franromero@ugr.es

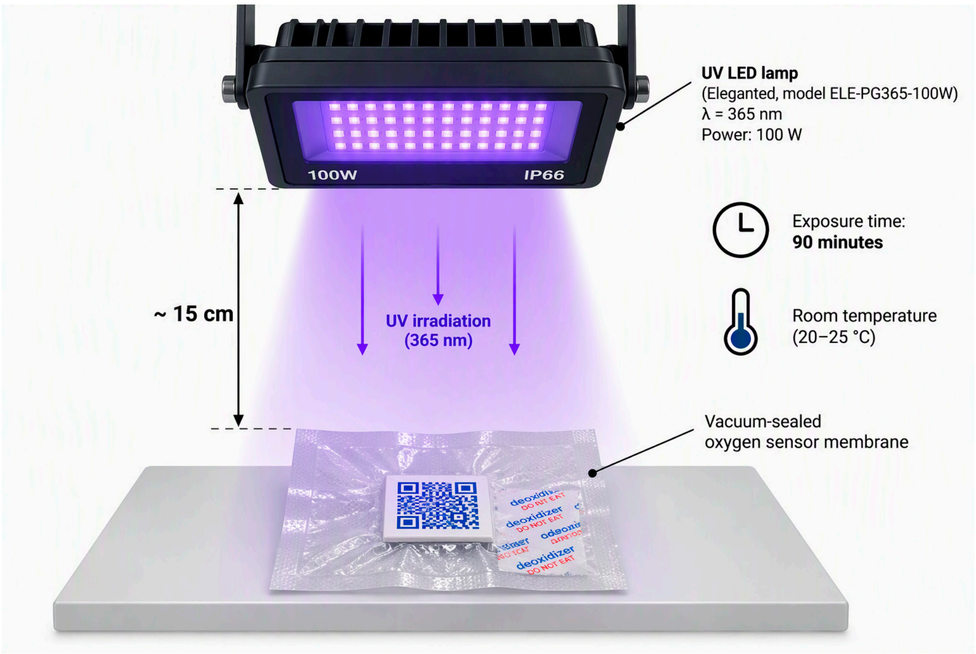

Figure S1. Setup used for the UV activation of the vacuum-sealed oxygen sensor: UV LED lamp (Eleganted, ELE-PG365-100W, λ = 365 nm, 100 W) placed approximately 15 cm above the package, with an exposure time of 90 min at room temperature.

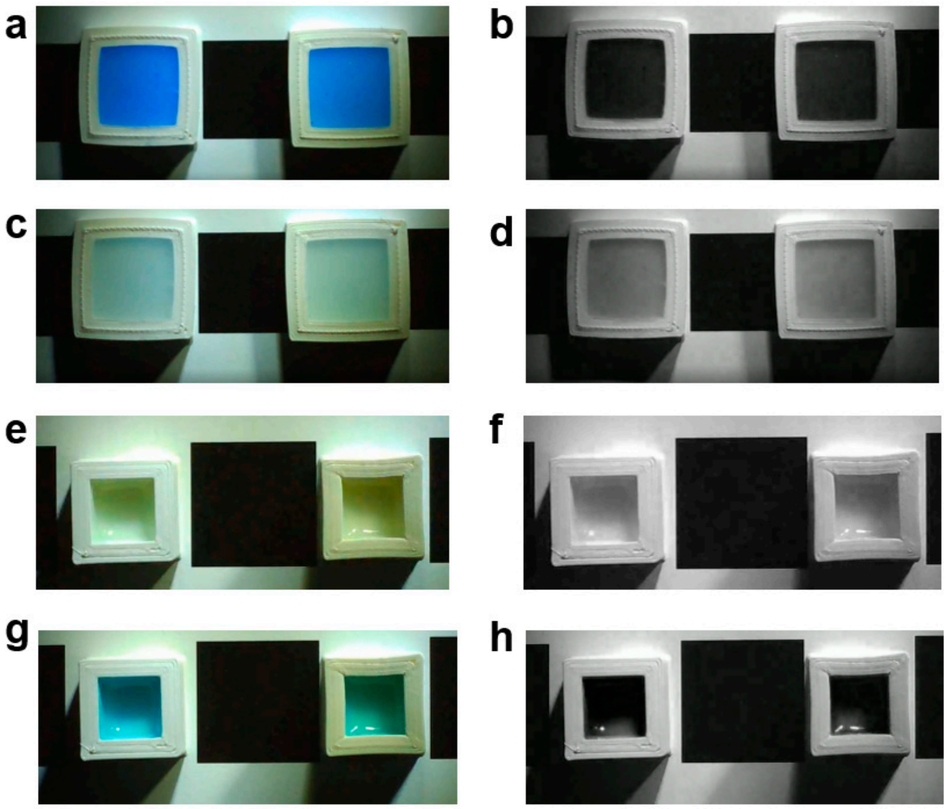

Figure S2. Representative images of the temperature and oxygen sensors in their different sensing states and the corresponding red-channel grayscale images. (a) Temperature sensor in its colored state ($T < T_{TH}$) and (b) its red-channel grayscale representation. (c) Temperature sensor in its colorless state ($T > T_{TH}$) and (d) its red-channel grayscale representation. (e) Photoactivated oxygen sensor immediately after exposure to ambient atmospheric conditions and (f) its red-channel grayscale representation. (g) Oxygen sensor after 60 min of exposure to ambient atmospheric conditions and (h) its red-channel grayscale representation.

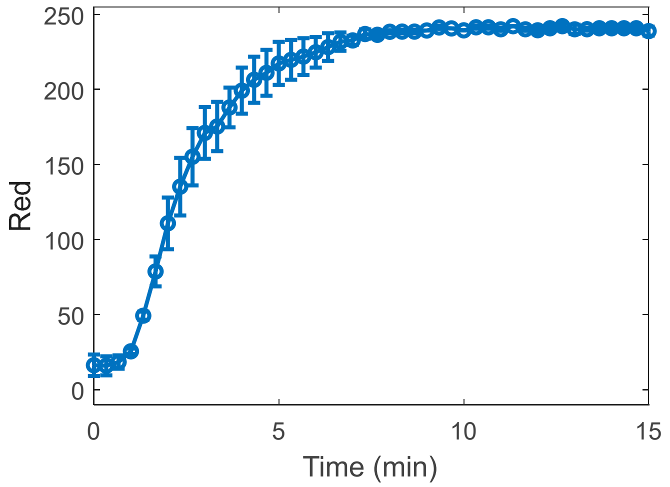

Figure S3. Red-channel intensity response of the temperature sensor (averaged for two sensors) after being transferred from a refrigerator (4 °C) to ambient temperature (27 °C).

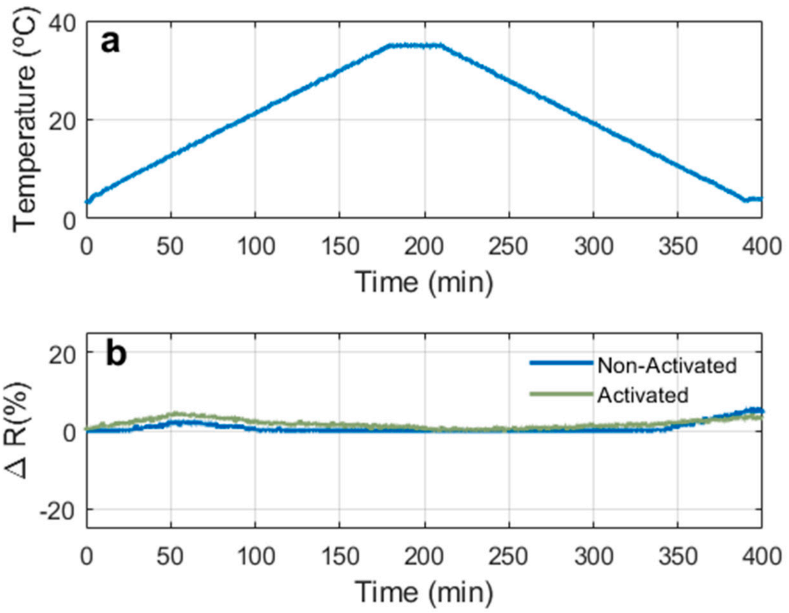

Figure S4. (a) Temperature profile applied to two vacuum-sealed oxygen sensors. (b) Relative variation of the red channel (ΔR, % of full scale) of a UV-activated (colorless) and a non-activated (blue) sensor during the heating–cooling cycle.

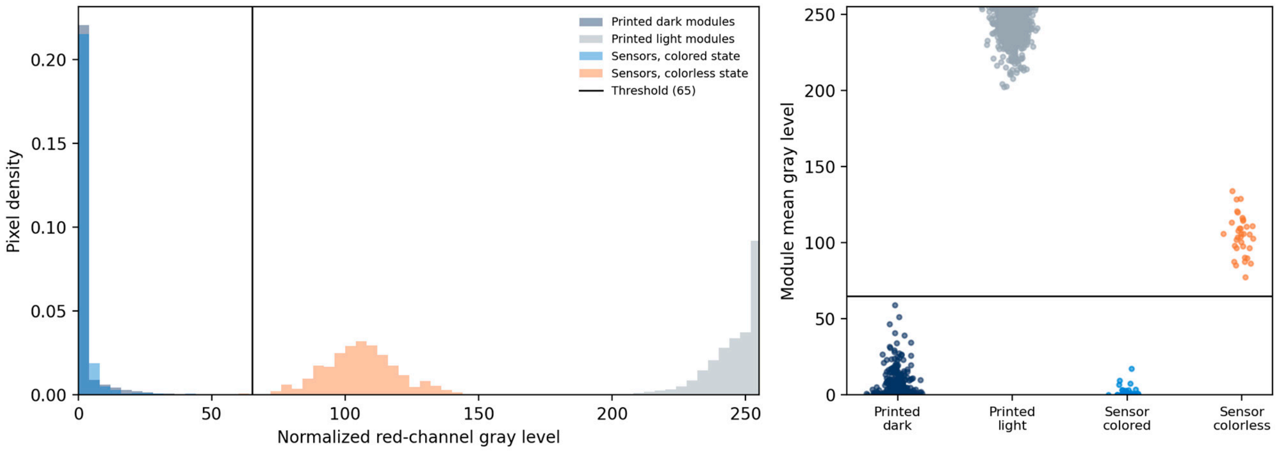

Figure S5. (a) Histogram of the normalized red-channel gray levels of the printed dark and light QR modules and of the sensing modules in their colored and colorless states, extracted from the four images of Figure 5 (ii). (b) Mean gray level of each module where the solid line indicates the binarization threshold (65/255).

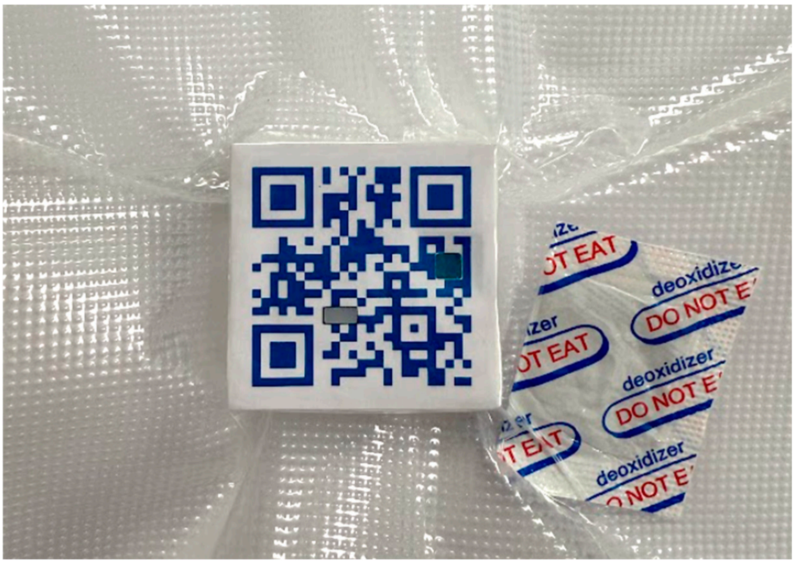

Figure S6. Vacuum-packed sensor module.

Video S1. Color change of the temperature sensor in RGB.
https://drive.google.com/file/d/1SRF_WOGEdWHpfI0aZu6ooP-S9PpLQWE5/view?usp=drive_link

Video S2. Color change of the temperature sensor in grayscale from red channel.
https://drive.google.com/file/d/1oCY6Lq1qgn5Zwv7tqUc7C4M4b8nEdrI5/view?usp=drive_link

Video S3. Color change of the oxygen sensor in RGB.
https://drive.google.com/file/d/1TpzJc6QEdecuM89xmHb-MsZsZkagHZ80/view?usp=drive_link

Video S4. Color change of the oxygen sensor in grayscale from red channel.
https://drive.google.com/file/d/17NbhYcudKUIhUnMr64OE8TWCtHyTCspJ/view?usp=drive_link

Video S5. Activated and non-activated oxygen sensors during a heating–cooling cycle between 4 and 35 °C in RGB.
https://drive.google.com/file/d/18NeMTVEK678AoouI-6JNe9j6RhyngVCq/view?usp=sharing

Video S6. Activated and non-activated oxygen sensors during a heating–cooling cycle between 4 and 35 °C in grayscale from red channel.
https://drive.google.com/file/d/1_Ah-Vn2ARiG2m1WK5Jku8Bhy8kndYjPv/view?usp=drive_link
